# Biomaterials and Tissue Biomechanics: A Match Made in Heaven?

**DOI:** 10.3390/ma10050528

**Published:** 2017-05-13

**Authors:** Amir A. Zadpoor

**Affiliations:** Department of Biomechanical Engineering, Faculty of Mechanical, Maritime and Materials Engineering, Delft University of Technology (TU Delft), Mekelweg 2, Delft 2628 CD, The Netherlands; a.a.zadpoor@tudelft.nl; Tel.: +31-15-278-1021; Fax: +31-15-278-4717

**Keywords:** biomaterials, tissue biomechanics, skeletal system, computational modeling, biomaterial discovery

## Abstract

Biomaterials and tissue biomechanics have been traditionally separate areas of research with relatively little overlap in terms of methodological approaches. Recent advances in both fields on the one hand and developments in fabrication techniques and design approaches on the other have prepared the ground for joint research efforts by both communities. Additive manufacturing and rational design are examples of the revolutionary fabrication techniques and design methodologies that could facilitate more intimate collaboration between biomaterial scientists and biomechanists. This editorial article highlights the various ways in which the research on tissue biomechanics and biomaterials are related to each other and could benefit from each other’s results and methodologies.

## 1. Introduction

Biomaterials and tissue biomechanics have traditionally been separate research areas with relatively little overlap in terms of methodological approach or research questions. The development of new biomaterials has primarily been a research area for researchers practicing applied chemistry, materials science, and biology. Tissue biomechanics, on the other hand, has generally borrowed its methods from applied physics and mechanics. There has therefore been relatively little interaction between the researchers of both communities, and research groups focusing on both areas simultaneously were rare, if not nonexistent. Recently, however, the boundaries between biomaterials and tissue biomechanics have started to fade and more research has been performed at the intersection of both fields. That is partially due to the maturity of the techniques developed by both communities that allows the focus to be at least partially shifted towards the interaction of those techniques with ones developed in other research communities. Moreover, certain characterization techniques such as atomic force microscopy (AFM) [1,2] and fabrication methods such as advanced additive manufacturing (3D printing) techniques [3] have become available that allow and call for more direct interactions between biomaterials and tissue biomechanics. That is why this special issue is devoted to the study of biomaterials and tissue biomechanics. The current editorial article aims at clarifying how biomaterials and tissue biomechanics research could be related to each other and what the current state of research at the intersection of both fields is. This editorial will specially focus on skeletal tissues and biomaterials that replace them either temporarily (to stimulate tissue regeneration) or permanently (as implants).

There are at least two ways through which the research on biomaterials and (tissue) biomechanics are related to each other. Firstly, it is generally assumed that the biomaterials replacing tissues should mimic their properties as much as possible [4]. One of the major goals of tissue biomechanics research is to characterize the mechanical and physical properties of healthy as well as pathological tissues. The outcomes of those measurements could be used as reference values for development of biomaterials that replaces the same tissues. Secondly, biomaterials that fulfil certain structural functions need to be tested under realistic loading conditions. The accurate estimation of detailed musculoskeletal loads experienced by tissues is therefore necessary. Since musculoskeletal loads cannot be often measured in vivo without resorting to invasive methods, the only realistic way for (patient-specific) estimation of musculoskeletal loads is to use biomechanical models. Moreover, given the fact that the forms (i.e., geometry) of skeletal tissues, particularly bone and muscle, follow their function (i.e., load history), there is also an inherent relationship between the (micro-scale) geometry of tissues and, thus, the biomaterials that replace them on the one hand and the musculoskeletal loading conditions experienced by the tissues on the other. In what follows, I will elaborate on the different ways in which biomaterials and tissue biomechanics research communities could contribute to each other’s research. 

## 2. From Biomaterial Development to Tissue Characterization

Design requirements are needed for the development of biomaterials that stimulate tissue regeneration or are used as permanent implants. In other words, one needs to know the ideal properties of the biomaterial one tries to develop. In many cases, it is assumed that the ideal properties for a biomaterial are those of the native tissue it replaces [4]. Those properties include not only mechanical properties such as elastic modulus, yield strength, and fatigue resistance, but also physical (mass transport) properties such as permeability and diffusivity [4]. It is important to note that this is not necessarily a perfect assumption, as the properties of the tissues in their equilibrium condition are not necessarily the best properties for stimulating tissue regeneration. Nevertheless, this tissue-mimicking principle is often used as the gold standard for deciding the ideal properties of biomaterials. The application of such a principle requires quantitative knowledge regarding the various properties of tissues. 

Experimental and computational studies in tissue biomechanics could provide the required data regarding the mechanical and physical properties of various types of tissues. From the experimental viewpoint, relatively simple mechanical testing procedures could be used to determine the large-scale properties of (skeletal) tissues. Those include compressive (confined and unconfined), tensile, shear, and cyclic (i.e., fatigue) loading procedures that are often used for mechanical characterization of tissue properties at the macro-scale. Indentation tests such as micro- and nano-indentation could be used for mechanical characterization of tissues at the micro- and nano-scales. Indentation-type testing could be performed either using nano-indenters or AFM machines. In the particular case of AFM, it is also possible to determine the mechanical properties of tissue constituents at the molecular level. In cartilage, for example, it is possible to distinguish between the mechanical properties of the collagen matrix and those of the glycosaminoglycans (GAGs) [5], and determine the content ratio of each constituent [6].

As far as mass transport properties such as permeability and diffusivity are concerned, basic fluid mechanics relationships such as Darcy’s law [7,8] as well as Fick’s law [9] could be in principle used to measure the permeability and diffusivity of different tissues and biomaterials in a relatively direct way. In addition, one could measure the mechanical and mass transport properties of tissues simultaneously, for example, through fitting bi- and multi-phasic models to the results of compression tests [10]. The latter approach ensures that the mechanical and mass transport properties are determined in a consistent manner. Ultimately, the mechanical properties determined using the experimental and computational approaches mentioned above could be used as reference values for the development of tissue-mimicking biomaterials. 

## 3. From Additively Manufactured Biomaterials to Quantitative/Functional Anatomy

Additive manufacturing offers the possibility to fabricate biomaterials and implants with arbitrarily complex geometries at macro- and micro-scales. From patient-specific implants [11] to AM porous biomaterials [12,13,14,15], multi-scale geometrical features could be used to improve the performance of both implants and biomaterials. However, quantitative information regarding the geometry of tissues and organs at various scales may be needed. For example, the shape of bones is often needed to design patient-specific orthopedic implants. Another example is the micro-architecture of trabecular bone that could be used for the design and AM of porous biomaterials [16]. 

Biomechanical studies of tissue and organs often require the same kind of quantitative data, which is why detailed models and data regarding the (functional) anatomy of the skeletal system has been generated within the tissue biomechanics community. In particular, statistical shape models (SSM) as well as statistical shape and appearance models (SSAM) of bones have been developed within the tissue biomechanics community [17]. SSMs are basically mathematical models that describe the average shape of bones within a statistical population [18]. In addition to the average shape, SSMs describe the main ways in which shapes are different from the average shape [18]. SSAMs contain additional info describing how the bone mineral density changes within bones. Since bone density is correlated with its mechanical properties [19,20,21], SSAMs also describe the distribution of tissue properties within bones and within statistical populations. The information regarding the bone shape could be used for the design of (patient-specific) implants, while the density distribution and associated mechanical properties could be used for the design of AM porous biomaterials. Similar types of quantitative information regarding other types of tissues, e.g., cartilage, are essential for other applications of additive manufacturing such as organ and tissue printing.

## 4. From Biomaterial Testing to Musculoskeletal Modeling

Mechanical characterization of biomaterials is the ultimate test to determine whether they could withstand the loads they will experience during their service life. Biomaterials have been often tested under simplistic loading conditions that do not necessarily represent the actual loading conditions. Not only are there no (patient-specific) aspects in the mechanical testing protocols used for biomaterials and implants, even the generic aspects of the loading conditions are not often adequately represented in the test protocols. That is partially due to the fact that the musculoskeletal loads experienced by tissues and, thus, the biomaterials or implants that replace them either temporarily or permanently are not always known. 

The experimental measurement of the musculoskeletal loads is next to impossible without using invasive techniques. Even when experimental techniques could be used for measurement of musculoskeletal loads [22,23,24], the number of patients are limited, individual muscle forces cannot be measured, and routine clinical application is infeasible. That is why biomechanical models are the only feasible options for the estimation of (patient-specific) musculoskeletal loads. There are at least three modeling approaches for the estimation of musculoskeletal loads. The first approach is based on using large-scale musculoskeletal models that use detailed information regarding bone shape and inertia, muscle parameters, muscle attachment sites, human movement patterns, and external forces to estimate the muscle loads as well as the joint reaction forces. The process of calculating musculoskeletal loads from human movement data and external force measurements using the abovementioned information regarding bones and muscles is called ‘inverse dynamics’. The loads predicted using large-scale musculoskeletal models [25,26,27] are often very detailed. For example, large muscles are usually represented by several regions with their specific lines of action. The magnitudes and directions of the forces associated with each of those regions are calculated in the inverse dynamics analysis. It is also possible to implement patient-specific aspects in large-scale musculoskeletal models [28]. 

The second types of models [29,30,31] is based on a simplified approach where multiple segments of the human body are represented by one single lumped mass. A few lumped masses therefore represent the entire human body. Masses are connected to each other with springs and dampers that represent the mechanical properties of different types of tissues. For example, hard and soft tissues may be separated and represented by different lumped masses [31,32,33,34]. Lower and upper extremity tissues could be also represented by different masses [32,34,35]. A few springs and dampers will then represent the connection between hard and soft tissues. The advantage of such simple mass-spring-damper models is that they are relatively easy to analyze, require limited data collection time, and have a limited number of parameters. Their disadvantage, however, is that it the loads they estimate are not very detailed and usually lack patient-specific aspects. 

The third approach is based on the solving the inverse problem of bone tissue adaptation. As we will see in the following section, the density distribution of bones could be at least partially described by the musculoskeletal loads they have experienced. Bone tissue adaptation models could be used to predict the density distribution resulting from a given load. If instead of mechanical loading, the density distribution is available (e.g., from computed tomography (CT) images), one could solve the inverse of the bone adaptation problem and find the musculoskeletal loads that have led to the measured density distribution [36,37,38,39]. Due to practical reasons such as limited computational power, the number of musculoskeletal loads that could be estimated using this latter approach is limited. Moreover, only average loads could be estimated, not the time history of the loads during specific movements. However, patient-specific aspects are relatively straightforward to implement.

Depending on the type of biomaterial or implant being tested and its service requirements, the loads estimated using one of the three abovementioned approaches could be used for designing mechanical testing protocols. 

## 5. From Rational Design of Biomaterials to Biomechanical Modeling of Tissue Adaptation

Powered by recent advances in additive manufacturing techniques, rational design of biomaterials has emerged as a recent trend where geometrical features at different scales are used for creating biomaterials and implants with novel properties and enhanced performance. Since the properties and performance of such biomaterials and implants are functions of the geometrical features and spatial distribution of materials (in multi-material AM), the shape and material composition need to be rationally designed. Rational design in this context refers to applying physical principles, analytical solutions, and computational models to predict what properties result from a given design of geometry and material distribution. Computational (as well as semi-analytical [40,41,42]) models are nowadays a mainstream tool for the study of numerous engineering [43,44,45] and biological [46,47,48] problems. It is therefore only natural that they be also used for the rational design of biomaterials (see e.g., [49,50]), particularly given the widespread availability of user-friendly computational packages that make this kind of technique accessible also to non-specialists. 

In the context of rational design of biomaterials, an interesting approach would be using computational models that simulate the biological process of tissue growth, adaptation, and remodeling. The tissue biomechanics community has been developing a large number of such computational models during the last few decades [51,52,53,54]. This approach is particularly meaningful for biomaterials that aim to replace biological tissues either temporarily or permanently. 

Of particular interest for bone-substituting biomaterials are bone tissue adaptation models. The micro-architecture of AM porous biomaterials could be rationally designed for any specific loading conditions simply by running the bone tissue adaptation algorithms that predict the micro-architecture of trabecular bone given the musculoskeletal loading conditions [55]. Based on such approaches, it is possible to design and additively manufacture patient-specific bone-substituting biomaterials. The (patient-specific) musculoskeletal loads determined by the approaches discussed in the previous section could be used in such a design approach.

Tissue biomechanics models could be also used in the design of patient-specific implants. Usually, more than one option is available for the design of patient-specific implants, particularly for complex bony reconstructions and trauma patients. Computational models developed in the tissue biomechanics community (see e.g., [56]), could predict what design results in the best stress/strain distribution and maximizes implant longevity. 

## 6. Conclusions

In this editorial article, we discussed some of the many different ways in which tissue biomechanics and biomaterials research communities could benefit from the tools and data generated in the other community. In the vast majority of cases, the tools developed in the tissue biomechanics community could guide the process of developing novel multi-functional biomaterials. The availability of advanced additive manufacturing techniques and the emerging concept of rational design of biomaterials and implants make such interactions between both communities even more meaningful and are expected to further intensify such interdisciplinary lines of research. A few examples of studies at the intersection of biomaterials and tissue biomechanics can be found in the present special issue. 
